# The Making of a Modern Hospital


**Published:** 1911-12-30

**Authors:** 


### THE MAKING OF A MODERN HOSPITAL.*

### The Ward Itself.

On few questions concerning hospital construction has there been more discussion than on the ideal ward. Even to-day, when the main essentials of such construction are generally agreed upon, there exists considerable difference of opinion with reference to the application of these essentials to the ward itself. The large number of so-called "ideal models" which are extant, and to the number of which every hospital adds another, shows very clearly that we have as yet arrived at no one type that can be called perfect. The full discussion of the various points which support such an opinion needs too much technicality to make it advisable to embark upon it in this series of articles. All that we may be permitted to do here is to give a summary of the generally accepted essentials which are now deemed to be imperatively necessary in a general ward of a hospital.

### Some Accepted Essentials.

Thus it is now recognised by all authorities that one of the most important preliminary details in ward construction is the "lie" of the building. The long axis of the ward should be north and south, so as to give the greatest amount of available sunshine, allowing for the room to be flooded with sunshine on the east side in the morning and on the west in the evening. This important preliminary was not generally admitted as an essential twenty-five years ago, and even now we see hospital wards the long axis of which is NE.—SW. or even directly east and west. Yet this essential is not absolute, especially in mountainous districts where a large amount of early morning sunshine and late afternoon light is cut off by the ranges, and where a ward in that position is exposed to the sweep of cold north-east winds. This is a point which architects who are responsible for the planning of hospitals in low-lying, mountain environed valleys sometimes lose sight of, with the result that patients in these model wards, whose short axis faces the east and west, are seriously inconvenienced by cold and draughts. Where such special circumstances obtain, however, it is obvious that some difference of arrangement is advisable. Generally all that is necessary is to afford the hospital sufficient shelter by pine plantations or by outbuildings which shield it effectively against cold north-easterly winds.

### What the British Architect Forgets.

Another essential of the ward is that it should be built in such a style that every available inch of floor space can be effectively illuminated by sunlight at some period of the day. Obviously therefore it is of the greatest importance that the amount of window space provided should be much larger than is usually the case. This additional provision of window space raises the question of heating—a matter which will be discussed later on. Here it may however be stated that British architects have not made as much use of the double window as they might have done. By providing double windows we at once safeguard the ward against undue cold, and moreover against outside noises. Only one who has been an inmate of a double windowed ward in a position where the adjacent streets are very noisy can rightly appreciate the vast difference which this simple device makes in the comfort of patients. There would be no necessity for expensive aluminium plating of the walls or for restrictions of traffic in the neighbourhood of the institutions if hospital architects paid more attention to the provision of double windows for their wards. Generally speaking the essentials of ward construction may, in the words of Depage, be epitomised as follows: decentralise, make allowance for ample air space, provide for adequate ventilation, be extravagant as far as natural lighting is concerned, choose a good site, simplify everything, allow nothing which is redundant and useless, use the best materials, and above all bear in mind the fact that the ward is designed for the use of sick people whose special comfort must be studied in the first instance.

As a preliminary let us consider the importance of one essential—the walls. Dampness in a hospital is a fault which presupposes carelessness on the part of the constructor. Such dampness may enter the building in four ways: during the actual construction of the walls, by using a surplus of water; by capillary suction later on when specially porous materials have been used; by condensation; and finally by direct infiltration. Against these possibilities the architect should guard by employing only the best material, by efficient drainage of the site, and by making adequate provision for overflow water.

Of late years much has been done in the way of experimentation to find some chemical means by which the action of damp on hospital walls can be prevented. We have carefully gone into the various methods employed and we have no hesitation in saying that none of them has any permanent value. The only way to ensure thorough dryness is to pay the most minute attention to the building, to the materials used, and to the prevention of the accumulation of moisture subsequently by physical means.

### The Nightingale Number of Beds.

How many beds should a ward contain? On this question, again, there is considerable diversity of opinion. The late Miss Nightingale favoured "less than twenty-two in each ward"; with that number, which may be taken as the modern average for a large hospital, administrative convenience is favoured and efficient overlooking ensured. The French hospitals, which are notoriously overcrowded, give an average of twenty-four, but lately the tendency has been to divide these larger wards

* Previous articles in this series appeared in The Hospital of October 7, 14, 21, 28, November 4, 18, and December 9.

into two smaller ones of twelve beds each. The Schaerbeek hospital in Belgium has twenty beds in each ward; the civil hospital at Havre only sixteen; the Heidelberg hospital only eleven; the cantonal hospital at Zurich only eight. The following interesting table gives the number of beds and the floor space, air space, and where available the surface lighting space for some of the newer institutions:—

Table Showing Dimensions of Wards.

| Hospital | Number of beds per ward | Surface per bed in square metres | Cubic air space per bed in cubic metres | Surface light in square metres |
|---|---|---|---|---|
| New York Hospital, 1890 ... | 21 | 10 | 50 | — |
| Zurich Hospital ... ... | 8 | 9·96 | 36 | 1·10 |
| Elizabeth Hospital, Vienna, 1890 [1] ... ... ... | 15 | 8·49 | 39 | — |
| Franz Joseph Hospital, 1892 [1] | 22 | 10·91 | 55 | — |
| New Hospital de la Pitie, Paris, 1902 ... ... | 20 | 10·27 | 46·60 | — |
| New Nuremberg Hospital. | 32 | 7·57 | 38·0 | — |
| Saint Eloi, Montpellier, 1890 | 28 | 10 | 66 | 1·92 |
| Virchow, Berlin, 1906 ... | 20 | 12·03 | 52 | — |
| Urban, Berlin, 1890 [2] ... | 32 | 9·18 | 47·20 | 1·26 |
| Schaerbek Hospital, 1906 [3] | 20 | 10·08 | 50 | 3·15 |
| Civil Hospital, Havre [2] ... | 14 | 9·0 | 49 | 1·70 |
| Johns Hopkins, Baltimore [2] | 24 | 12 | 60 | 2·60 |
| Civil Hospital, Bernburg ... | 15 | 8 | 40 | — |
| Civil Hospital, St. Denis ... | 16 | 11·57 | 67 | 3·50 |
| Holy Ghost, Rome [4] ... | 220 | 7·18 | 110·0 | — |
| Eppendorf, Hamburg ... | 30 | 7·30 | 37·0 | 2·56 |
| St. Thomas, London ... | 28 | 10·80 | 49 | 2·90 |
| Blackburn Infirmary ... | 8 | 10·80 | 50 | 3·04 |
| New portion Guy's Hospital | — | 10·26 | 44 | 3·71 |
| Lariboisière, Paris [2] ... | 32 | 10·90 | 56· 0 | 1·34 |
| New Surgical Clinic, Gray's Inn Road ... ... | 20 | 10·80 | 54 | 3·40 |

1 The beds are badly placed, two between each window.
2 Two beds between each window.
3 An excellent hospital.
4 This is an old hospital given by way of comparison. The ventilation of the huge wards is very imperfect, notwithstanding the large amount of cubic air space provided, owing to the fact that the walls of the ward are nearly 50 feet high.

The Twelve-bed Ward.

There is at the present moment a general agreement that wherever possible the number of beds in a ward should be curtailed. Most authorities favour the twelve-bedded ward. Questions of administration and nursing will of course weigh very largely in deciding this question. For example, English authorities consider foreign hospitals to be much understaffed. In Germany three nurses, one probationer and one male attendant are estimated as requisite in a twenty-four bedded ward; in some of the Belgian hospitals there is only one nurse to every twenty-four patients.

With regard to air space, modern wards allow an average of 50 cubic metres and most countries where hospital construction is regulated by State decrees prescribe forty cubic metres as the irreducible minimum. Much more important than cubic content, however, is efficient ventilation, and when this test is applied it is a lamentable fact that many modern hospitals which are otherwise up-to-date are sadly deficient.

Here we may step aside for a moment to point out the foolishness of trying to secure cubic content by increasing the height of the ward walls—a favourite endeavour of older architects. No advantage whatever is obtained by raising the walls above fourteen feet, and the Belgian Hospital Commission, which went very fully into this question, reported strongly in favour of a maximum height of four and a half metres.

Depage on Wall Space.

With regard to floor space, considerable differences are seen in the various hospitals constructed in modern times. Messrs. Depage and Vandervelde, in their monograph on hospital construction, conclude as follows, after discussing the various modifications suggested: "It seems to us that a surface of twelve square metres per bed is quite sufficient. With the ward walls four and a half metres high, this gives us 54 cubic metres of air space. This is ample and most convenient in all respects." With this opinion most modern authorities will agree, though in England the tendency is to allow rather more surface per bed. It is essential that each bed should be placed between two windows, and that there should be adequate "circulating space" between each patient, while sufficient space should also be left to allow for a locker alongside each bed.

The questions regarding the size of bed, the length of wall, and the general dimensions of the ward are best considered in another article, when we shall give various models of what may be styled perfect wards. Such a comparison will serve a more useful purpose than an academic enumeration of the various points of importance, for it will enable the reader to judge the actual designs and test them by the essentials already given.

